# Various Options for Covalent Immobilization of Cysteine Proteases—Ficin, Papain, Bromelain

**DOI:** 10.3390/ijms26020547

**Published:** 2025-01-10

**Authors:** Marina G. Holyavka, Svetlana S. Goncharova, Valeriy G. Artyukhov

**Affiliations:** 1 Biophysics and Biotechnology Department, Voronezh State University, 1 Universitetskaya Square, 394018 Voronezh, Russiaartyukhov@bio.vsu.ru (V.G.A.); 2 Bioresource Potential of the Seaside Territory Laboratory, Sevastopol State University, 33 Studencheskaya Street, 299053 Sevastopol, Russia

**Keywords:** ficin, papain, bromelain, covalent immobilization

## Abstract

This study explores various methods for the covalent immobilization of cysteine proteases (ficin, papain, and bromelain). Covalent immobilization involves the formation of covalent bonds between the enzyme and a carrier or between enzyme molecules themselves without a carrier using a crosslinking agent. This process enhances the stability of the enzyme and allows for the creation of preparations with specific and controlled properties. The objective of this study is to evaluate the impact of covalent immobilization under different conditions on the proteolytic activity of the enzymes. The most favorable results were achieved by immobilizing ficin and bromelain through covalent bonding to medium and high molecular weight chitosans, using 5 and 3.33% glutaraldehyde solutions, respectively. For papain, 5 and 6.67% glutaraldehyde solutions proved to be more effective as crosslinking agents. These findings indicate that covalent immobilization can enhance the performance of these enzymes as biocatalysts, with potential applications in various biotechnological fields.

## 1. Introduction

Biocatalysts are widely used in biotechnology, medicine, pharmaceutical, and tanning industries due to their high substrate specificity and ease of production. However, the application of enzymes on an industrial scale is limited by their low reuse rates. Additionally, the stability of some enzymes during biochemical reactions can be compromised. These limitations can be addressed through enzyme immobilization. The methods of enzyme immobilization are well studied, and ongoing research continues to provide more detailed descriptions of the mechanisms involved in these processes. There is currently no universal method for immobilizing proteolytic enzymes, as each method has its own advantages and disadvantages. The most effective immobilization method for each enzyme is determined experimentally and depends on the research objectives. To achieve greater stability of the preparations, the structure of the biocatalyst should be carefully considered. Studying the specifics of enzyme immobilization on different carriers enables the development of new continuous industrial technologies for producing target products through heterogeneous catalysis. However, it is important to recognize that for each enzyme used in industrial processes, the most suitable carrier, conditions, and immobilization methods should be selected.

Proteases are crucial in regulating various biological processes in plants, including pathogen and pest recognition and the initiation of effective defense mechanisms. Over 110 latices from different plant families are known to contain at least one proteolytic enzyme, most commonly from the cysteine and serine endopeptidase families [1]. So, latex is a significant source of plant proteases and has a long history of use in traditional medicine and various industries [2,3]. The cysteine protease family consists of six main groups, with most latex cysteine proteases belonging to the Papain family (C1). Papain (EC 3.4.22.2) is the proteolytically active component found in the latex of the tropical papaya fruit *Carica papaya*. As the most extensively studied member of the cysteine protease family, papain exhibits endopeptidase, amidase, and esterase activities. The enzyme is synthesized as an inactive precursor [4,5] and is localized within the latex of the laticifer system in the plant [6]. Extracts from the papaya plant have been used to treat warts, corns, piles and yaws, nerve pain, infected wounds, and malignant tumors [7] and to combat parasitic infections and gastrointestinal nematodes such as ascarids, tapeworms, whipworms, and hookworms [8]. Additionally, papain-containing ointments are employed in wound debridement to remove necrotic tissue from chronic wounds and burns [9,10].

Ficin (EC 3.4.22.3) is the primary proteolytic enzyme found in Ficus latex. It shares broad similarities with papain in terms of specificity, and its amino acid composition closely resembles that of papain. The amino acid sequence around ficin’s active site is highly similar to the corresponding site in papain [6]. Proteolytic extracts from Ficus latex are used in serology to unmask antigens [11]; to treat various conditions, including ulcers, bile secretion disorders, psoriasis, anemia, piles, jaundice, nasal and oral hemorrhages, and blood-related diseases; and as antidysenteric, purgative, and emetic agents [12]. Historically, ficin has attracted interest due to its ability to digest gastrointestinal nematodes, although it has not been widely adopted as a medical treatment for nematode infections [6]. Ficin has also demonstrated effectiveness against biofilms of *Staphylococcus aureus* [13] and *Staphylococcus epidermidis* [14] and oral pathogens, and it plays a role in wound healing [15].

Bromelain (EC 3.4.22.4) is an endopeptidase derived from *Ananas comosus*. It is recommended for treating burn wounds and is a key ingredient in the drug NexoBrid © (marketed as Debrase in the USA) [16,17,18]. Due to its low toxicity, bromelain is an effective tool for managing chronic inflammatory diseases. It exhibits immunomodulatory properties and accelerates tissue repair by depolymerizing intercellular structures and altering vascular permeability [19,20]. Bromelain is also used in cosmetology for acne treatment [21,22] and has demonstrated anti-biofilm activity [23]. Like papain and ficin, bromelain is a monomeric protein, meaning it consists of a single polypeptide chain [24].

Enzyme immobilization enables the recovery and reuse of the biocatalyst [25], reducing or preventing product contamination by protein molecules [26]. The use of an immobilized biocatalyst simplifies reactor design and allows for easier control of the reaction, as the biocatalyst can be filtered out to stop the process [27]. When properly planned and executed, enzyme immobilization can enhance certain enzyme properties, such as activity, specificity, or selectivity [28]. Additionally, some immobilization techniques can be combined with enzyme purification [29]. Research suggests that targeting specific areas of the protein for immobilization can help maintain its stability under denaturing conditions, ensuring consistent enzyme performance during operation and storage. However, immobilization can also result in reduced enzyme activity [30].

Chitosan, derived from the deacetylation of chitin, has garnered significant interest as a cost-effective and versatile biomaterial for immobilizing various enzymes across fields such as medicine [31,32], wastewater treatment [33], agriculture [34], and biotechnology [35,36]. This polyaminosaccharide, composed of β-(1–4)-linked D-glucosamine and *N*-acetyl-D-glucosamine units, offers several desirable properties, including its natural origin, antibacterial activity, non-toxicity, ease of modification, biodegradability, and low cost [37,38]. The presence of primary amino and hydroxyl groups in chitosan enhances interactions between the biopolymer and enzymes, enabling the use of straightforward immobilization methods like adsorption and covalent bonding [39,40]. Covalent bonding, in particular, provides a stronger attachment of the enzyme to the carrier, reducing the risk of leaching in aqueous environments. Additionally, it can improve the stability of the biocatalyst and modify its catalytic properties [41].

The covalent immobilization method, whether through single- or multiple-point bonding, creates covalent links between the functional groups on the enzyme and the support surface. Enzymes immobilized through multipoint covalent attachment typically exhibit greater operational and storage stability compared to those immobilized by physical adsorption. This increased stability is likely due to the covalent bonds between the enzyme and the support, which help rigidify certain areas of the protein, enhancing its overall stability [42,43].

Enzyme immobilization is considered an effective technique for enhancing enzyme stability for several reasons [44,45]. One key factor is that multipoint covalent attachment between the enzyme and the support is expected to increase the enzyme’s rigidity. This stabilizing effect is more pronounced when the spacer arm is short and the support is rigid. Additionally, the stabilizing effect tends to increase as more enzyme–support linkages are formed, leading to a more rigid overall three-dimensional enzyme structure [27,46,47,48,49]. This rigidity helps maintain the relative positions of all involved groups within the spacer distance during any conformational changes. This process, known as enzyme multipoint covalent attachment, is facilitated by extended enzyme–support reaction times [50,51,52]. Although achieving this is complex, the potential benefits in terms of enzyme stabilization are substantial [44]. When using monomeric enzymes, if the primary cause of inactivation is related to structural distortion, research has shown that enzyme stability under various conditions correlates with the number of enzyme–support bonds [50,51,52]. In these situations, enzyme stability improves as the enzyme–support reaction time increases. After reaching a certain maximum level, stability remains constant over reasonable time periods [53].

Another widely used method for stabilizing enzymes is crosslinking their molecules, also referred to as carrier-free immobilization. In this approach, the enzyme itself serves as the carrier, avoiding the benefits and drawbacks associated with external carriers and allowing the creation of a pure enzymatic system. The crosslinking process utilizes bi- or multifunctional reagents, known as linkers, to connect enzyme molecules into three-dimensional cross-linked enzyme aggregates. Surface chemicals can also serve as potential linkers for forming these enzyme aggregates [54,55]. Glutaraldehyde is the most commonly used bifunctional agent due to its low cost and wide availability. It facilitates crosslinking between the free amino groups on the enzyme surfaces, primarily lysine residues and glutaraldehyde [56,57]. A significant advantage of this method is that crosslinking produces cross-linked enzyme aggregates (CLEAs), which offer a carrier-free immobilization approach that integrates both enzyme purification and immobilization into a single process [58,59,60]. Covalent immobilization of enzymes without a carrier matrix involves forming covalent bonds between enzyme molecules using a crosslinking agent, where the enzyme itself serves as the carrier. A key consideration is that the immobilization process should not interfere with the functional groups essential for the enzyme’s catalytic activity [61]. Cross-linked enzyme aggregates (CLEAs) represent a modern and flexible carrier-free immobilization technique. CLEAs are particularly appealing due to their simplicity and robustness, eliminating the need for purification, and they typically exhibit high catalytic specificity and selectivity, enhanced operational and storage stability, and good reusability [62]. This method is advantageous as it simplifies the process by removing extraneous materials and allows the combined use of crude enzyme mixtures [63].

However, the immobilization of CLEAs presents several challenges. One major issue is their low mechanical strength, especially in aqueous solutions, as their consistency resembles that of gelatin [64]. Additionally, controlling the size of the aggregates is difficult [65] due to the high reactivity of the bifunctional crosslinking agents used in this method. Because of CLEAs’ low mechanical resistance, reactor designs should accommodate low shear stress conditions. Another challenge arises from the size of CLEAs in large-scale applications. Small aggregates can complicate filtration and centrifugation processes after reactions, while larger particles can cause diffusion issues, potentially reducing the biocatalyst’s efficiency [65].

Amino-glutaraldehyde groups tend to react more readily with other amino-glutaraldehyde groups rather than with single amino groups. Furthermore, two glutaraldehyde molecules linked to a single amino group do not easily react with other amino-glutaraldehyde groups, which helps to prevent excessive protein modification [66,67]. Therefore, the optimal concentration of glutaraldehyde should be high enough to allow modification with a single molecule but not so high that it results in the formation of complex amino-glutaraldehyde–glutaraldehyde structures.

Glutaraldehyde is also a primary agent used to activate supports for enzyme immobilization [66]. It reacts with primary amines to create covalent crosslinks. The process of enzyme immobilization with glutaraldehyde typically begins with the enzyme adsorbing onto the support through ionic or hydrophobic interactions, followed by the formation of covalent bonds that enhance enzyme stability. Therefore, higher concentrations of glutaraldehyde can offer multiple interaction sites for the enzyme, potentially leading to improved outcomes.

Currently, the use of glutaraldehyde as a crosslinker for immobilizing enzymes is a topic of active discussion in the literature. The advantages of glutaraldehyde include (i) its ability to act as a short crosslinker, forming brief spacer arms that provide the enzyme with sufficient movement space [68] by reacting with itself and the support; (ii) its effectiveness in activating supports to achieve consistent and uniform covalent interactions [69]; and (iii) its good reactivity across a relatively wide pH range, which allows for beneficial inter- or intramolecular modifications [66]. In terms of toxicity, glutaraldehyde is less toxic compared to some other cross-linkers used in covalent binding, such as isocyanates, carbodiimides, divinyl sulfone, and diazo benzidine, which also may not offer the same level of crosslinking effectiveness as glutaraldehyde [70].

The strategies for covalent enzyme immobilization vary based on the type of functional group used for the coupling reaction and factors such as selectivity, loading capacity, immobilization time, and the activity/stability of the final immobilized preparation. However, no single approach can address all these criteria effectively, highlighting the challenge of developing a versatile process that is broadly compatible with protein surface residues and common linker functional groups [71].

In light of the above, the objective of this work was to optimize the covalent immobilization methods for cysteine proteases—ficin, papain, and bromelain—using chitosan as the support and crosslinking the enzyme molecules with glutaraldehyde.

## 2. Results

### 2.1. Developing a Method for Covalent Immobilization of Cysteine Proteases on a Chitosan Matrix

Covalent immobilization relies on the formation of covalent bonds between the enzyme and the carrier using a crosslinking agent, resulting in strong bonds that enhance the enzyme’s stability. This method enables the production of enzyme preparations with specific and controlled properties.

**Figure 1:**
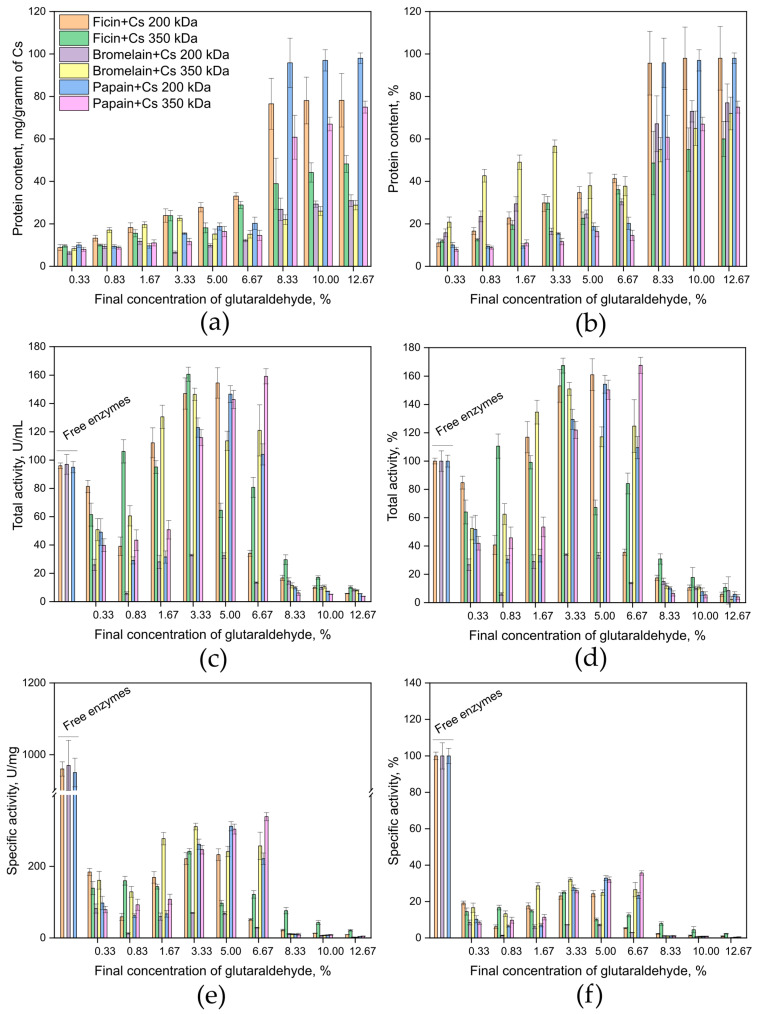
Protein content in mg/g of carrier (**a**) and % (**b**); total catalytic activity in units/mL of solution (**c**) and % (**d**); specific catalytic activity in units/mg of protein (**e**) and % (**f**) for the samples of ficin, papain, and bromelain immobilized on a medium molecular weight chitosan matrix (200 kDa) and a high molecular weight chitosan matrix (350 kDa). Protein content, total catalytic activity, and specific catalytic activity of ficin’s, papain’s, and bromelain’s solutions before enzyme immobilization were taken as 100%.

An analysis of the protein content in the obtained samples via the Lowry method [72] showed that the highest amount of ficin, papain, and bromelain was observed when they were immobilized by covalent binding to medium and high molecular weight chitosans using 8.33, 10, and 12.67% glutaraldehyde solutions. In this case, the sorption efficiency (based on protein content) was, respectively, not less than 95 and ~50–60% for ficin, not less than 95 and ~60–75% for papain, and ~67–77 and ~55–75% for bromelain. In addition, during covalent immobilization on high molecular weight chitosan, bromelain content was quite high when using 1.67 and 3.33% glutaraldehyde, with sorption efficiencies ranging from 50 to 55% (Figure 1a,b).

The total activity of ficin (units per mL of solution) to azocasein as a substrate [73] was higher when immobilized by covalent binding to medium molecular weight chitosan using 3.33 and 5% glutaraldehyde solutions. Compared to the free form, the activity of the immobilized biocatalyst increased by 53 and 61%, respectively. When immobilized on high molecular weight chitosan, the highest activity of ficin was observed with a 3.33% glutaraldehyde solution, increasing by 67% compared to the free enzyme. Papain activity was higher when immobilized by covalent binding to medium molecular weight chitosan using a 5% glutaraldehyde solution, achieving 154% of the free enzyme’s activity. During covalent immobilization on high molecular weight chitosan, the highest activity was detected with a 6.67% glutaraldehyde solution, amounting to 167% of the free enzyme’s activity. Bromelain showed activity values satisfactory for industrial use when covalently bound to medium molecular weight chitosan using glutaraldehyde concentrations ranging from 0.33 to 5%, achieving 25 to 30% of the free enzyme’s activity. Typically, industry replaces the immobilized enzyme when the residual activity is between 50 and 10% of the initial activity [74].

During covalent immobilization on high molecular weight chitosan, bromelain activity was highest when using glutaraldehyde concentrations from 1.67 to 6.67%, reaching 120 to 150% of the free enzyme’s activity (Figure 1c,d).

Ficin samples demonstrated acceptable specific activity when covalently immobilized to a chitosan matrix using 3.33 and 5% glutaraldehyde solutions, achieving slightly more than 20% of the specific activity of the free enzyme. High specific activity during ficin immobilization on high molecular weight chitosan was detected using a 3.33% glutaraldehyde solution, reaching 25% of the free enzyme’s specific activity. Papain showed higher specific activity when immobilized by covalent binding to medium molecular weight chitosan using a 5% glutaraldehyde solution, achieving 33% of the free enzyme’s specific activity. During covalent binding to high molecular weight chitosan, the highest specific activity of papain was observed with a 6.67% glutaraldehyde solution, reaching 36% of the free enzyme’s specific activity. Bromelain exhibited high specific activity when covalently bound to medium molecular weight chitosan using 0.33, 3.33, and 5% glutaraldehyde solutions, achieving 7 to 8% of the free enzyme’s specific activity. When covalently bound to high molecular weight chitosan, bromelain showed the highest specific activity with glutaraldehyde concentrations from 1.67 to 6.67%, achieving 25 to 30% of the free enzyme’s specific activity (Figure 1e,f).

The optimal ratio of protein content (mg per g of carrier), total activity (units per mL of solution), and specific activity (units per mg of protein) was achieved during the immobilization of ficin and bromelain by covalent binding to medium and high molecular weight chitosans using 5 and 3.3% glutaraldehyde solutions, respectively, and during immobilization of papain by covalent binding to medium and high molecular weight chitosans using 5 and 6.67% glutaraldehyde solutions, respectively.

**Figure 2:**
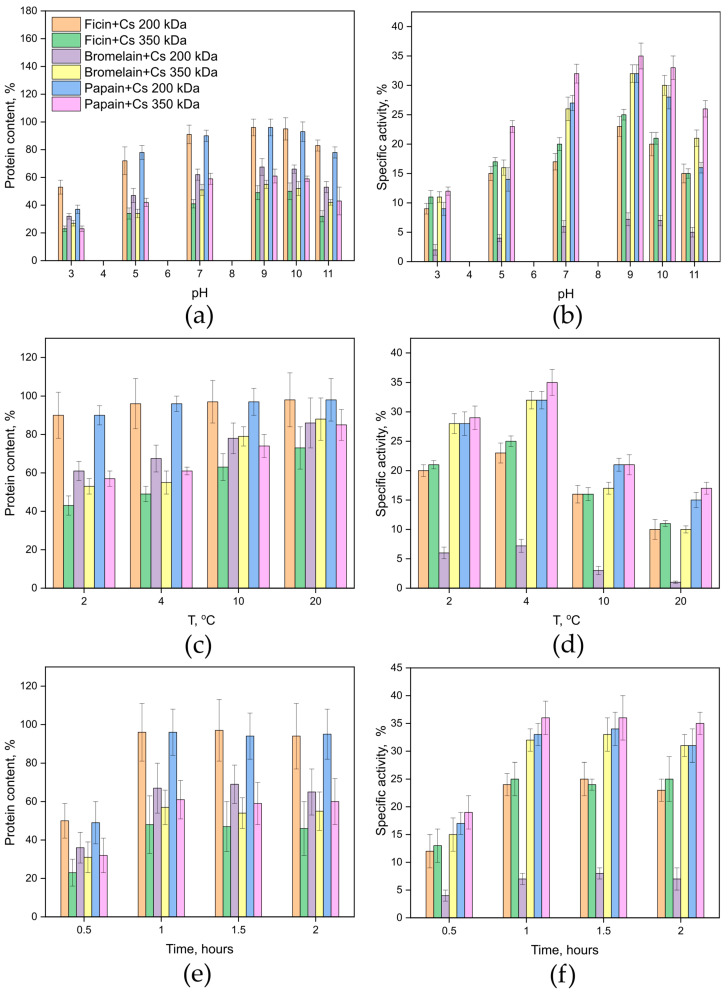
Protein content (**left**) and specific catalytic activity (**right**) for the samples of ficin, papain, and bromelain immobilized on chitosan matrix at various pH ((**a**,**b**): temperature 4 °C, time of immobilization—1 h), temperature ((**c**,**d**): pH 9.7, time of immobilization—1 h), and time of immobilization process ((**e**,**f**): temperature 4 °C, pH 9.7). Protein content and specific catalytic activity of ficin’s, papain’s, and bromelain’s solutions before enzyme’s immobilization were taken as 100%.

Next, for the named (optimal in our opinion) concentrations of glutaraldehyde, we optimized the temperature and pH of the medium for the covalent immobilization of cysteine proteases on the chitosan matrix. It has been established that it is advisable to use buffers with a pH of 9–10 for covalent immobilization of enzymes. At pH 9–10, we observe the highest protein content and the highest percentage of activity retention with the covalent immobilization of ficin, papain, and bromelain on chitosan (Figure 2a,b), which has a completely logical theoretical explanation. Glutaraldehyde reacts reversibly with amino groups across a broad pH range (≥pH 3.0), with minimal reversibility between pH 7.0 and 9.0. Lysine ε-amino groups have a pKa > 9.5, but the small percentage of unprotonated amines at lower pH levels is sufficient for reacting with glutaraldehyde. Excessive crosslinking at pH > 10 may increase steric hindrance for large protein substrates.

We also showed that the optimal temperature for covalent immobilization of cysteine proteases on chitosan is 4 °C. At 2 °C, the protein content and activity of the immobilized samples did not differ significantly from those obtained at a temperature of 4 °C, but since 4 °C is easier to achieve in laboratory conditions than 2 °C, we opted for 4 °C. When the temperature was increased to 10 and 20 °C (Figure 2c,d), the amount of immobilized enzyme increased, but its activity decreased significantly, probably due to denaturation processes caused by the action of glutaraldehyde.

It was found that the optimal incubation time for covalent immobilization of ficin, papain, and bromelain on chitosan is 1 h (Figure 2e,f). When the enzymes were incubated with the carrier for 0.5 h, the immobilization efficiency for both protein content and activity was approximately 50% of that for 1 h of incubation. When the immobilization time was increased to 1.5 and 2 h, the immobilization efficiency did not increase compared to 1 h.

### 2.2. Developing a Method for Covalent Immobilization of Cysteine Proteases Without a Carrier Matrix

**Figure 3:**
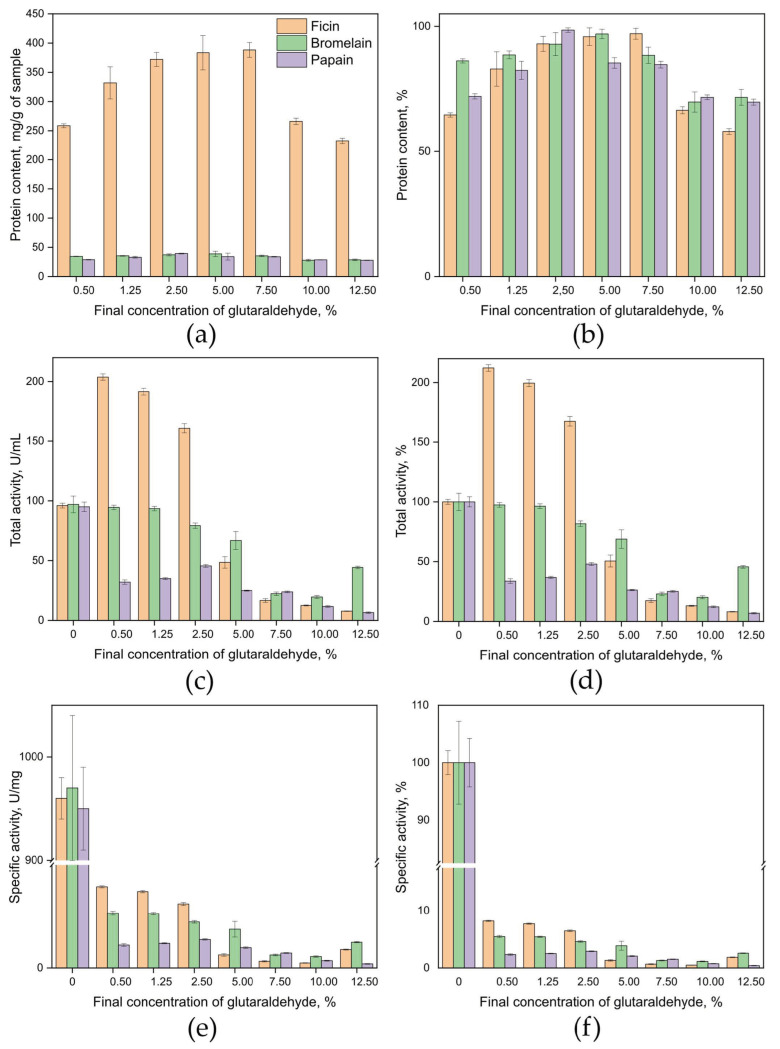
Protein content in mg/g of sample (**a**) and % (**b**); total catalytic activity in units/mL of solution (**c**) and % (**d**); specific catalytic activity in units/mg of protein (**e**) and % (**f**) for the samples of ficin, papain, and bromelain covalently immobilized without a carrier matrix. Protein content, total catalytic activity, and specific catalytic activity of ficin’s, papain’s, and bromelain’s solutions before enzyme immobilization were taken as 100%.

Protein content analysis of the samples revealed that ficin immobilized with 7.5% glutaraldehyde had the highest protein retention, achieving a binding efficiency of 97%. Papain demonstrated higher protein content when immobilized with 2.5% glutaraldehyde, with a binding efficiency of 98%. Bromelain samples showed substantial protein retention when immobilized with 5% glutaraldehyde, achieving a 97% binding efficiency (Figure 3a,b).

The total activity of ficin (units per mL of solution) was highest when immobilized with 0.5% glutaraldehyde, resulting in a 112% increase in activity compared to the free enzyme. Papain exhibited greater activity when immobilized with 2.5% glutaraldehyde, maintaining 48% of the activity of the free enzyme. Bromelain retained a high level of activity when immobilized with 0.5% glutaraldehyde, preserving 97% of the activity of the free enzyme (Figure 3c,d). The specific activity of all three enzymes during immobilization by the CLEAs method had low values and did not exceed 3–8% at all concentrations of glutaraldehyde used by us. (Figure 3e,f).

The optimal balance of protein content (mg per g of enzyme) and total activity (units per mL of solution) was identified for ficin and bromelain immobilized with 0.5% glutaraldehyde. For papain, the most effective concentration of the crosslinking agent was found to be 2.5%.

## 3. Discussion

Chemical immobilization, which involves forming new covalent bonds between the enzyme and the carrier, is the most common method for creating industrial biocatalysts. This method requires the use of functional groups that are not essential for the enzyme’s activity, such as –NH_2_, –COOH, –SH, or –OH. However, placing the enzyme at a distance of one covalent bond from the carrier can create steric hindrance that affects the catalytic process. To address this, enzymes are often separated from the carrier using a spacer (crosslink), which can be a bifunctional or polyfunctional agent such as cyanogen bromide, hydrazine, sulfuryl chloride, or glutaraldehyde.

**Figure 4:**
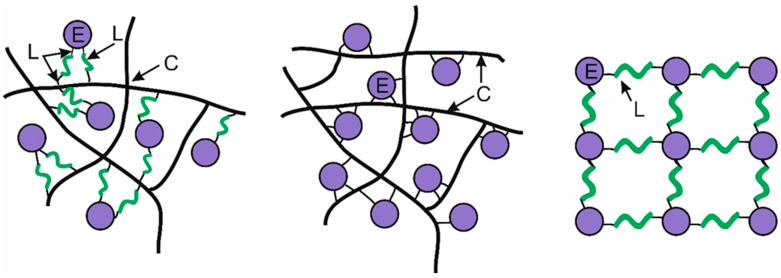
Variants of covalent immobilization of enzymes: the enzyme molecule (E, purple color), the carrier (C, black color), and the crosslinking reagent (L, green color).

Despite the variety of components and complexities involved, chemical immobilization fundamentally relies on three key elements: the enzyme molecule (E), the carrier (C), and the crosslinking reagent (L). The immobilization structures can be represented as follows (Figure 4):

- E–C–L, where the enzyme is linked to both the carrier and the crosslinking reagent;
- E–C, where the enzyme is directly attached to the carrier;
- E–L, where the enzyme is linked to the crosslinking reagent.

The E–C configuration, which lacks a crosslinking agent, poses significant steric challenges for large substrates, including proteins. Therefore, in our work, we did not use this approach for immobilizing cysteine proteases.

When using monomeric enzymes, if the primary cause of inactivation is related to structural distortion, research has shown a correlation between enzyme activity and stability under various conditions and the number of enzyme–support bonds [51,52]. Typically, enzyme stability improves with increased enzyme–support reaction time, and it stabilizes after reaching a certain maximum, remaining constant over reasonable periods [50,53,75]. However, this pattern does not apply to all monomeric enzymes; for instance, some cysteine proteases exhibit different behavior.

The results indicate that the cysteine residue responsible for the catalytic activity of ficin is exposed to the medium [76]. This exposure allows it to participate in the immobilization process on supports activated with thiol-reactive groups, leading to complete enzyme inactivation. For example, this made it impossible to use vinyl sulfone-activated supports for immobilizing this enzyme extract [77]. However, protecting the cysteine residue before immobilization and deprotecting it afterward successfully addressed this issue.

Ficin was immobilized on glyoxyl agarose and then cross-linked with glutaraldehyde. In both cases, stability increased with longer incubation times, but after reaching a maximum, extending the enzyme–support or crosslinking reaction times led to a significant decrease in enzyme stability [78,79,80]. This was unexpected, as one would anticipate that the number of enzyme–support bonds would either increase or remain stable over time and that multipoint covalent attachment would result in a more rigid, not less stable, enzyme structure [50,51,52].

Our experiments showed similar results. During covalent immobilization on chitosan with glutaraldehyde as the crosslinking agent, the amount of cross-linked enzyme increased as the concentration of the crosslinking agent rose from 0.33 to 8.33%. The optimal proteolytic activity was achieved when ficin and bromelain were immobilized on chitosan with glutaraldehyde concentrations of 3.33 to 5%, while papain showed the best results with concentrations of 5 to 6.67%.

Despite its success in enzyme immobilization, glutaraldehyde’s chemistry remains controversial [81]. Although its simple structure belies its complex behavior and reactivity in aqueous solutions [82], the reaction mechanism between glutaraldehyde and the enzyme’s amino groups may involve Schiff base formation, Michael addition, and nucleophilic substitution [63]. At lower glutaraldehyde concentrations, fewer aldehyde groups are formed, leading to lower immobilization efficiency. Conversely, particularly at concentrations above 2.5%, immobilization efficiency decreases when using the CLEA method. This reduction is likely due to steric hindrance caused by the excessive number of crosslinking points [83].

To identify the mechanisms of covalent immobilization of ficin, papain, and bromelain on the chitosan matrix and without carrier via the CLEAs method, we used FTIR.

**Figure 5:**
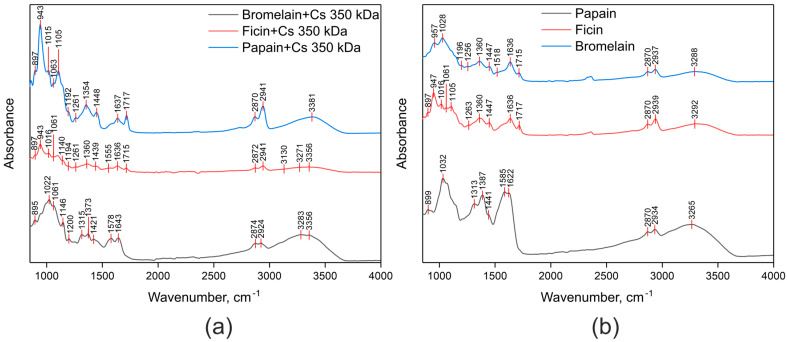
FTIR spectra of enzymes covalently immobilized on chitosan and prepared with 8.33% glutaraldehyde (**a**) and CLEAs prepared with 5% glutaraldehyde (**b**).

Figure 5a shows the FTIR spectra of enzymes covalently immobilized on chitosan, prepared with 8.33% glutaraldehyde. These data confirm successful immobilization, as characteristic bands of all reagents are present: chitosan pyranose ring vibrations in the region of 895–1146 cm^−1^, amide I bands of enzymes at 1636–1643 cm^−1^, and partially unbound glutaraldehyde C=O stretching at 1715–1717 cm^−1^. The Schiff base C=N peaks, appearing at 1615–1650 cm^−1^ [84,85], overlap with the enzyme’s amide I bands, while the low intensity of amide II bands (νN-H + δC-N) for both chitosan and enzymes indicates that enzyme crosslinking occurs primarily through amino groups. Figure 5b shows the FTIR spectra of CLEAs prepared with 5% glutaraldehyde. As seen in the figure, the intensity of the amide II bands (νN-H + δC-N) is significantly lower compared to the amide I bands (νC=O). This observation confirms that enzyme crosslinking occurs primarily through available amino groups.

**Figure 6:**
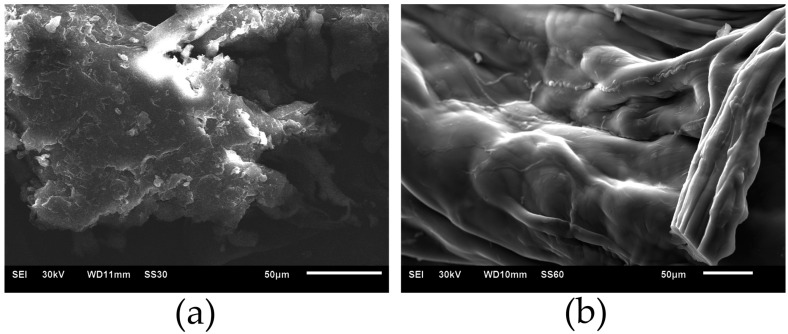
SEM images of surface of chitosan (**a**) and ficin–chitosan complex (**b**).

SEM data confirm changes in the surface of chitosan after the immobilization of cysteine proteases on it. Initially, chitosan has a scaly surface (Figure 6a), but after the immobilization of ficin on it, the protein–polysaccharide complex exhibits a smooth surface (Figure 6b).

Glutaraldehyde can react with various protein functional groups, including amine, thiol, phenol, and imidazole [63], due to the nucleophilic nature of the most reactive amino acid side chains. Studies have reported the reactivity of aldehydes (at pH levels ranging from 2.0 to 11.0) with amino acids such as lysine, tyrosine, tryptophan, phenylalanine, histidine, cysteine, proline, serine, glycine, glycylglycine, and arginine [86,87,88]. These investigations have ranked the reactive groups of amino acids in decreasing order of reactivity as follows: ε-amino, α-amino, guanidinyl, secondary amino, and hydroxyl groups.

Avrameas and Ternynck [89] observed that glutaraldehyde either did not react with the amine function of the guanidinyl group (arginine) or that more reactive groups in protein molecules obscured arginine’s reactivity with glutaraldehyde. Okuda et al. [90] found that glutaraldehyde reacted with thiol groups only in the presence of a primary amino group. Additionally, glutaraldehyde reacts reversibly with amino groups across a broad pH range (≥pH 3.0), except between pH 7.0 and 9.0, where reversibility is minimal [90]. For this reason, we used buffers with a pH of 9–10 for the covalent immobilization of cysteine proteases.

Crosslinking proteins, whether to a carrier (solid support) or between protein molecules (carrier-free), typically involves the ε-amino group of lysine residues [56,57,91]. These unprotonated amino groups are highly reactive as nucleophiles [92]. It is important to note that lysyl ε-amino groups have a pKa (acid dissociation constant) greater than 9.5, but even the small percentage of amines in their unprotonated form at lower pH can react with glutaraldehyde. This reaction then shifts the acid–base equilibrium toward deprotonation, allowing further reactions to occur.

Most proteins contain multiple lysine residues, which are usually located on the protein surface and exposed to the aqueous medium due to the polarity of the amine group. Additionally, lysine residues are generally not part of the catalytic site, enabling moderate crosslinking without compromising the protein’s conformation [93] and biological activity [94]. As previously mentioned, glutaraldehyde exists in several forms in aqueous solution, and all of these forms may be reactive toward the lysine residues (ε-amino groups) of proteins.

**Figure 7:**
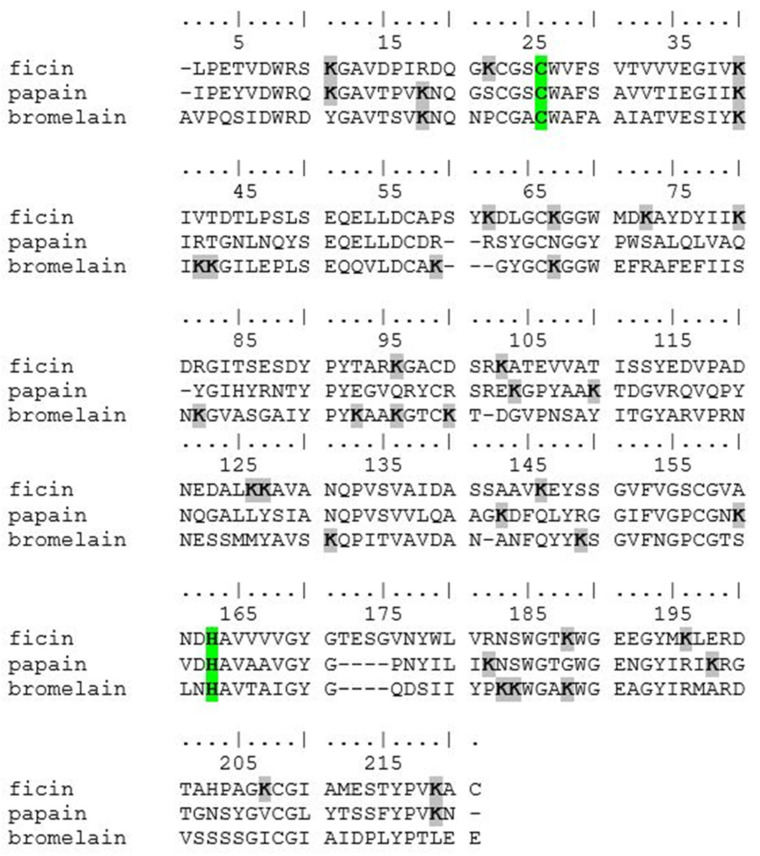
Alignment of amino acid sequences of ficin, papain, and bromelain. Active sites (Cys and His) highlighted in green, and Lys residues highlighted in gray.

**Figure 8:**
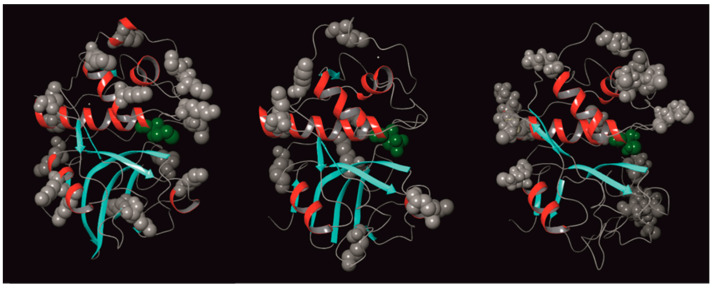
Lys residues in ficin, PDB ID: 4YYW (**left**), papain, PDB ID: 9PAP (**center**), and bromelain, PDB ID: 1W0Q (**right**) molecules: the atoms of the amino acid residues in their composition are shown as balls. Cys from active site highlighted in green, and Lys residues highlighted in gray.

It is noteworthy that, despite the structural similarities among cysteine proteases, a higher concentration of glutaraldehyde is necessary to achieve maximum activity in immobilized papain samples compared to ficin and bromelain. This discrepancy can be explained by the number of lysine residues present in each enzyme: papain contains 10 lysine residues, while bromelain and ficin have 15 and 16 lysines, respectively (Figure 7). Additionally, in all three enzymes, the lysine residues are positioned at a significant distance from the active site (Figure 8).

Multipoint covalent attachment of enzymes typically leads to more stable biocatalysts when the primary cause of inactivation is conformational changes due to environmental conditions, essentially when inactivation is linked to structural distortion. However, if the main cause of enzyme inactivation is something else or shifts to something else following the distortion caused by immobilization (as in the case of oxidation of the catalytic cysteine), simply rigidifying the enzyme’s structure does not necessarily enhance its operational stability. In fact, the increase in enzyme–support linkages could expose the catalytic group more to the medium, thereby accelerating inactivation. This scenario is observed in sulfhydryl proteases, where prolonged enzyme–support interactions in the presence of oxygen result in a less stable biocatalyst compared to shorter incubation times. However, if the oxidation of the cysteine residue is prevented, the enzyme exhibits higher activity [76]. To protect the active site from oxidation, cysteine proteases were immobilized in a 0.04 M cysteine solution.

Furthermore, the reaction of glutaraldehyde with aminoglycan chitosan not only facilitates crosslinking between polymer chains [94,95] but also enhances the mechanical strength of the carrier, preventing it from dissolving in acidic aqueous environments due to its cationic nature [35].

In conclusion, the chemical interactions between glutaraldehyde and proteins are not fully understood, and the mechanisms behind protein crosslinking remain speculative. It is likely that multiple mechanisms contribute to the reaction between glutaraldehyde and proteins. Given that glutaraldehyde can exist in various forms even under specific and controlled conditions, several reaction pathways may occur simultaneously. Enzyme immobilization can be successfully achieved under a broad range of conditions, which should be selected based on the desired outcomes. These conditions are often determined through trial and error, as the process of insolubilization depends on a delicate balance of factors, including the enzyme’s nature [96,97], the concentrations of both the enzyme [98] and the reagent [96], the pH [99] and ionic strength [100] of the solution, temperature [101], and reaction time [102]. Notably, the lysine content of the enzyme plays a significant role in its crosslinking efficiency with glutaraldehyde [96,97].

As previously mentioned, the concentrations of both the enzyme and glutaraldehyde should be carefully optimized to produce water-insoluble enzyme derivatives through crosslinking. Low concentrations of either component can lead to intramolecular crosslinking, as the glutaraldehyde functional groups are more likely to react with the same enzyme molecule [98]. Therefore, it is crucial to select conditions that promote intermolecular crosslinking between enzyme molecules, avoiding undesirable intramolecular bonds [103,104]. Broun [96] noted that the amount of crosslinking agent used significantly influences the degree of crosslinking. He observed that low glutaraldehyde concentrations may not form enough crosslinks to precipitate the enzyme, while higher concentrations can create a sufficiently tight structure by excluding water molecules, thus rendering the enzyme derivative insoluble. Chui and Wan [105] found that enzymatic activity decreases with increasing glutaraldehyde concentration, as excessive crosslinking can distort the enzyme’s structure, particularly its active site. This distortion can reduce substrate accessibility and binding, thereby diminishing biological activity. Additionally, the ratio of enzyme to glutaraldehyde should be carefully balanced to optimize results [88].

The reaction of glutaraldehyde with enzymes, leading to both soluble and insoluble products, has been widely studied and shown to be pH-dependent [106]. The optimal pH for glutaraldehyde-induced insolubilization varies between proteins. Researchers found that the pH at which the fastest insolubilization occurred for proteins like BSA, soybean trypsin inhibitor, lysozyme (EC 3.2.1.17), and papain (EC 3.4.22.2) closely matched their respective isoelectric points (pIs). In contrast, the insolubilization of active chymotrypsin (EC 3.4.21.1) was most rapid at pH 6.2, despite its pI of 8.6, and, for chymotrypsinogen A, the optimum was at pH 8.2, with a pI of 9.5. These observations suggest that the charge of the protein plays a crucial role in the intermolecular crosslinking necessary for insolubilization, with maximal crosslinking occurring when repulsive charges are minimal [99].

Moreover, Tomimatsu et al. [100] and Broun [96] noted that lower ionic strength in the reaction medium accelerated the crosslinking of chymotrypsin. Additionally, when selecting the pH for immobilization, the reactivity of aqueous glutaraldehyde should also be considered, as most immobilizations are carried out in a neutral to slightly alkaline pH range.

Broun reported the impact of temperature and reaction time on enzyme insolubilization [96]. In the early studies of enzyme immobilization, reactions were typically conducted at low temperatures (around 4 °C) to preserve fragile molecules. However, this approach often necessitated extended reaction times for the immobilization process to be effective [97].

The catalytic activity of water-insoluble enzyme derivatives created with multifunctional reagents like glutaraldehyde can vary significantly [99,101,107]. This variation is influenced not only by the amount of crosslinking reagent used during the insolubilization process but also by several other factors [108].

The effectiveness of glutaraldehyde as a crosslinking agent is due to its multicomponent nature, with various forms present in equilibrium in the reagent solution at a given pH. Despite extensive research, the precise molecular composition of glutaraldehyde solutions and the most reactive component remain subjects of debate. Consequently, the exact mechanism by which glutaraldehyde reacts with protein amino groups is not fully understood. It appears that no single mechanism accounts for all glutaraldehyde–protein crosslinking reactions. All known forms of glutaraldehyde can react with and crosslink proteins, resulting in a diverse range of conjugates. Achieving efficient insolubilization requires careful control of reaction conditions tailored to the structural variability of different enzymes. Despite the often unavoidable partial inactivation of enzymes due to chemical modification, most enzyme derivatives maintain sufficient catalytic activity and stability for reuse. Further research is needed to clarify the exact structure of these cross-linked products.

If we compare the methods of covalent immobilization of cysteine proteases with the methods of their physical immobilization on the chitosan matrix (Table 1), we can conclude that the main advantage of chemical immobilization is the high stability of the resulting complex, while its proteolytic activity is usually lower than in the case of adsorption on chitosan [23,85,109], entrapment in chitosan gels [110], and complex formation with chitosan nanoparticles [111,112].

## 4. Materials and Methods

### 4.1. Materials

In our study, we focused on ficin (23.4 kDa), papain (23 kDa), and bromelain (23.8 kDa) (Sigma, St. Louis, MO, USA). Azocasein (Sigma, St. Louis, MO, USA) was used as the substrate for hydrolysis. The immobilization carriers were medium molecular weight chitosan (200 kDa) and high molecular weight chitosan (350 kDa) (Bioprogress, Moscow, Russian Federation).

### 4.2. Method for Covalent Immobilization on a Chitosan Matrix

An amount of 20 mL of enzyme solution at the following concentrations (which provide an optimal protein–chitosan ratio, Figure S1) (for ficin, 4 mg/mL in 0.05 M glycine buffer, pH 10.0; for papain, 5 mg/mL in 0.05 M glycine buffer, pH 9.0; for bromelain, 2 mg/mL in 0.05 M Tris-glycine buffer, pH 9.0) and 10 mL of glutaraldehyde at various concentrations (1, 2.5, 5, 10, 15, 20, 25, 30, and 38%) were added to 1 g of chitosan [116]. Thus, The final concentrations of glutaraldehyde in the system were 0.33, 0.83, 1.67, 3.33, 5.0, 6.67, 8.33, 10.0, and 12.67%. To protect the active site from oxidation, the immobilization of cysteine proteases was carried out in a 0.04 M cysteine solution.

The resulting mixture was gently stirred intermittently and incubated for 1 h at 4 °C. The suspension was then centrifuged at 1500 g for 10 min. After incubation, the resulting preparation was dialyzed against 0.05 M Tris-HCl buffer, pH 7.5, using a cellophane membrane with a pore size of 25 kDa, until the eluate no longer contained any protein (monitored using an SF-2000 spectrophotometer (Spectr, Saint Petersburg, Russian Federation) at λ = 280 nm). As a result, the immobilized samples were free of unbound enzymes.

### 4.3. Method for Covalent Immobilization of Enzymes Without a Carrier Matrix

An amount of 5 mL of glutaraldehyde at various concentrations (1, 2.5, 5, 10, 15, 20, and 25%) was added to 5 mL of enzyme solution (at the following concentrations selected earlier: for ficin, 20 mg/mL in 0.05 M glycine buffer, pH 10.0; for papain, 2 mg/mL in 0.05 M glycine buffer, pH 9.0; for bromelain, 2 mg/mL in 0.05 M Tris-glycine buffer, pH 9.0). Thus, the final concentrations of glutaraldehyde in the system were 0.5, 1.25, 2.5, 5.0, 7.5, 10.0, and 12.5%. To protect the active site from oxidation, the immobilization of cysteine proteases was carried out in a 0.04 M cysteine solution.

The resulting solution was left in a Petri dish at 4 °C until it dried out. After incubation, the preparation was dialyzed against 0.05 M Tris-HCl buffer, pH 7.5, using a cellophane membrane with a pore size of 25 kDa, until the eluate no longer contained any protein (monitored using an SF-2000 spectrophotometer at λ = 280 nm). As a result, the immobilized samples were free of unbound enzymes.

### 4.4. Method for Determining Proteolytic Activity of Enzymes

The proteolytic activity of the enzymes was determined using azocasein as a substrate. The proteolytic activity was evaluated by measuring the absorbance of the proteolytic fragments of azocasein at 410 nm, as described in [73], with modifications. Briefly, the sample was suspended in 200 μL of buffer (50 mM Tris-HCl, pH 7.5), mixed with 800 μL of azocasein solution (0.5% in the same buffer), and incubated for 30 min at 37 °C. Then, 800 μL of the 5% trichloroacetic acid (TCA) solution was added, followed by the removal of the precipitated unhydrolyzed azocasein after 10 min incubation at 4 °C by centrifugation (3 min 13,000 rpm). Supernatant (1200 μL) was mixed with 240 μL of 1 M NaOH solution, and the optical density was measured at 410 nm. In the reference sample, the TCA was added prior to the enzyme. The protein content in the immobilized enzyme preparations was determined using the Lowry method [72]. The unit of catalytic activity was defined as the amount of enzyme that hydrolyzed 1 μM of substrate per minute under the experimental conditions.

The efficiency of enzyme immobilization was expressed in two ways: by protein content (binding efficiency), as a percentage of the amount of enzyme in the final preparation relative to the amount in the initial solution (considered as 100%), and by specific catalytic activity, as a percentage of the retained specific proteolytic activity of the enzyme after immobilization compared to the specific catalytic activity of the enzyme in the initial solution (considered as 100%) [75].

### 4.5. Fourier-Transform Infrared Spectroscopy

Fourier-transform infrared spectroscopy (FTIR) with attenuated total reflectance (ATR) was used for the structural characterization of immobilized enzymes. Spectra were recorded using a Bruker Vertex 70 instrument (Bruker Corporation, Billerica, MA, USA) with a Fourier transducer in the 850–4000 cm^−1^ range. Each measurement consisted of 32 scans per cycle, with a total of 4 cycles. The samples were analyzed in powder form.

### 4.6. SEM

The surface morphology of the chitosan and enzyme–chitosan complexes was studied by scanning electron microscopy using a JSM-6510LV scanning electron microscope (Jeol Ltd., Tokyo, Japan) in SEI mode. Prior to photography, a 10 nm gold layer was sprayed onto the samples.

### 4.7. Statistical Processing of Research Results

All experiments were conducted with a minimum of 8 replicates. Statistical analysis of the data was performed using the Stadia 8.0 Professional software package (available at http://protein.bio.msu.ru/~akula/Podr2~1.htm, accessed on 14 June 2024). The statistical significance of differences between control and experimental values was assessed using Student’s t-test, assuming a normal distribution of all parameters. The results are presented as the mean ± confidence interval, with statistical significance set at *p* < 0.05.

## 5. Conclusions

In light of data from other studies and the complexity of the interactions between glutaraldehyde, enzyme molecules, and chitosan chains, we aimed to optimize the covalent immobilization methods for cysteine proteases—ficin, papain, and bromelain—on chitosan and through crosslinking enzyme molecules with glutaraldehyde. The best results were achieved when ficin and bromelain were covalently bound to medium and high molecular weight chitosans using 5 and 3.33% glutaraldehyde solutions, respectively. For papain, 5 and 6.67% glutaraldehyde solutions were more effective as crosslinking agents. Cross-linked enzyme aggregates (CLEAs) were found to be less effective compared to the covalent immobilization of cysteine proteases on chitosan. This may be due to the incomplete elimination of autolysis processes or the more pronounced oxidation of cysteine at the enzyme’s active site by glutaraldehyde in the absence of a carrier. Additionally, steric hindrance could be more significant when crosslinking enzyme molecules without a polymeric carrier.

Therefore, the following conditions are recommended for the covalent immobilization of ficin, papain, and bromelain on chitosan:

1. The optimal enzyme-to-chitosan ratios are 8:100 for ficin, 10:100 for papain, and 4:100 for bromelain.
2. Since glutaraldehyde reacts reversibly with amino groups across a broad pH range (≥pH 3.0), with minimal reversibility between pH 7.0 and 9.0, it is advisable to use buffers with a pH of 9–10 for covalent immobilization. Lysine ε-amino groups have a pKa > 9.5, but the small percentage of unprotonated amines at lower pH levels is sufficient for reacting with glutaraldehyde. Excessive crosslinking may increase steric hindrance for large protein substrates.
3. Final concentrations of glutaraldehyde in the immobilization system should be in the range of 3.33–6.67%.
4. To protect the active site from oxidation, immobilization of cysteine proteases should be carried out in a 0.04 M cysteine solution.
5. The immobilization process should be conducted at a temperature of 4 °C.
6. To ensure that no unbound enzyme remains, the final step in sample processing should involve dialysis against a 0.05 M tris-HCl buffer, pH 7.5, using a cellophane membrane with a pore size of 25 kDa.

## Figures and Tables

**Table 1:** Methods of protease immobilization on chitosan: their advantages (+) and disadvantages (-).

Adsorption [10,23,29,85,109]	+	(1) simplicity (2) the possibility of modifying the carrier, giving it any configuration and the required porosity (3) the structure of the enzyme changes to a lesser extent than with covalent binding (4) it is often possible to simultaneously solve the problem of purifying the enzyme since the binding of the protein to the carrier is, in many cases, quite specific
	-	(1) low strength of enzyme binding to the carrier, and when immobilization conditions change, enzyme desorption and contamination of reaction products may occur (2) use of sufficiently large amounts of catalyst in the immobilization process (3) lack of general recommendations that allow one to make the correct choice of carrier and optimal conditions for immobilization of a specific enzyme in advance
Gel entrapment [110,113]	+	(1) simplicity (2) applicability for immobilization of not only single enzymes but also multienzyme complexes (3) uniform distribution of the enzyme in the volume of the carrier (4) the enzyme is not actually attached to the carrier, due to which there are no steric hindrances that arise during covalent or electrostatic binding of the enzyme to the polymer (5) the enzyme is protected from many unfavorable environmental factors, including from inactivation due to bacterial contamination since large bacterial cells cannot penetrate the fine-pored polymer matrix (6) the ability to create immobilized samples of any geometric configuration (spherical particles, films, etc.)
	-	(1) this method is unsuitable for immobilization of enzymes acting on poorly soluble substrates (2) diffusion limitations for high-molecular substrates, which is a significant disadvantage for proteases, the substrates of which are usually high-molecular proteins
Complexation with nanomaterials [111,112]	+	(1) a fairly narrow size distribution (2) a large surface area per unit of activity of the immobilized enzyme (3) virtually no steric hindrances arising from covalent or electrostatic binding of the enzyme to the polymer
	-	(1) the difficulty of obtaining stable forms of carriers (2) relatively low stability both in vitro and in vivo
Chemical immobilization [114,115]	+	(1) strong and irreversible bond between the enzyme and the carrier (2) significant stabilization of the enzyme molecule (3) the possibility of creating enzymes with controlled properties (in particular substrate specificity)
	-	(1) complexity of the technique (2) high cost (3) use of fairly large quantities of catalyst in the immobilization process

## Data Availability

The data presented in this study are available on request from the corresponding author. The data are not publicly available as they are part of the continuous study.

## Supplementary Materials

The following supporting information can be downloaded at https://www.mdpi.com/article/10.3390/ijms26020547/s1.
