# Supplementary material for: Various Options for Covalent Immobilization of Cysteine Proteases—Ficin, Papain, Bromelain
Source: Int J Mol Sci. 2025 Jan 10;26(2):547. doi: 10.3390/ijms26020547 (PMC11764635; doi:10.3390/ijms26020547)
Supplement: Supplementary file 1 [file ijms-26-00547-s001.zip › ijms-3395884-supplementary.pdf]

Various Options for Covalent Immobilization of Cysteine Proteases – Ficin, Papain, Bromelain

Marina G. Holyavka, Svetlana S. Goncharova and Valeriy G. Artyukhov

Supplementary materials

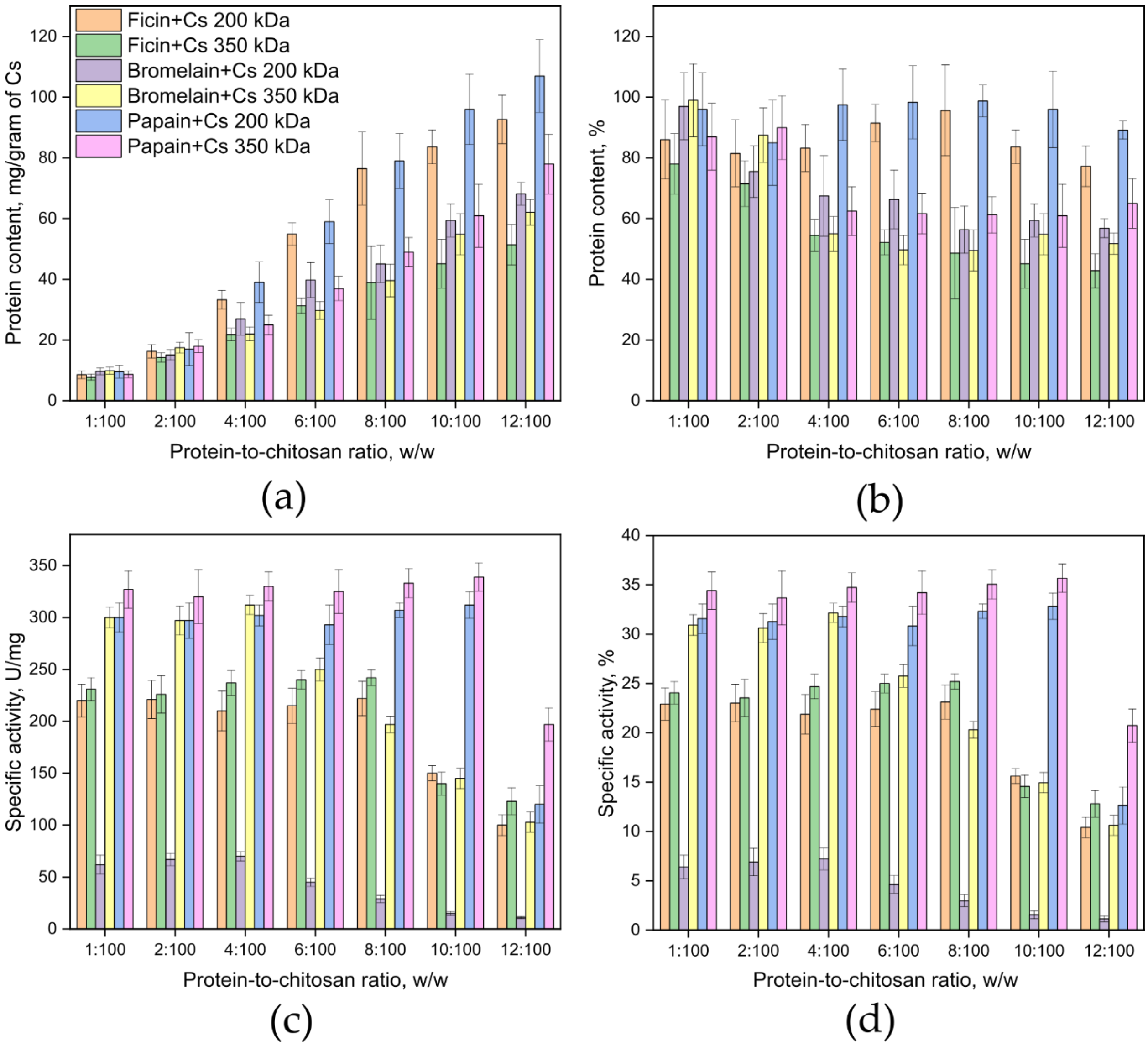

Figure S1. Protein content in mg/g of carrier (a) and in % (b), specific catalytic activity in units/mg of protein (c) and in % (d) for the samples of ficin, papain, and bromelain immobilized on a medium molecular weight chitosan matrix (200 kDa) and on a high molecular weight chitosan matrix (350 kDa). Protein content and specific catalytic activity of ficin's, papain's, and bromelain's solutions before enzyme's immobilization were taken as 100%.
